# Evaluation of activity and function before and immediately after the provision of a microprocessor knee in individuals with transfemoral amputation

**DOI:** 10.1097/PXR.0000000000000449

**Published:** 2025-04-09

**Authors:** Silvia Caggiari, Tim Randell, Chantel Ostler, Alex Dickinson, Peter Worsley

**Affiliations:** 1 Skin Sensing Research Group, School of Health Sciences, University of Southampton, Southampton, United Kingdom; 2 The Dorset Prosthetic Centre, Royal Bournemouth Hospital, Bournemouth, United Kingdom; 3 Portsmouth Enablement Centre, Portsmouth Hospitals NHS Trust, St Marys Community Health Campus, Portsmouth, United Kingdom; 4 People Powered Prosthetics Research Group, University of Southampton, Southampton, United Kingdom; 5 Bioengineering Science Research Group, Faculty of Engineering and Physical Sciences, University of Southampton, Southampton, United Kingdom

**Keywords:** microprocessor knees, lower limb amputation, physical activity, mobility, function, activity monitor

## Abstract

**Background:**

In many cases, individuals with lower limb amputation become less active because of impaired balance and stability and increased risk of falling. Microprocessor knees (MPKs) have been shown to reduce the risk of falls, improve balance, and increase function, evaluated with self-reported scales and questionnaires. This study aims at investigating whether the patient-reported improvements are reflected in objective physical activity (PA) parameters estimated from actimetry sensors and assess the short-term provision of an MPK.

**Study Design:**

Transfemoral amputee patients (n=29) undertaking an MPK trial at 2 prosthetic centers in the South of England were recruited for this study. Self-reported and functional test outcomes (Activities Balance Confidence, Reintegration of Normal Living Index, Prosthesis Evaluation Questionnaire scores, and 2-min walk test) were obtained before and after (4 weeks) the provision of the MPK. Activity levels were recorded over 7 consecutive days pre- and post-MPK.

**Results:**

Self-reported scores and function test outcomes showed a general improvement in most of the patients after the provision of the MPK, with a statistically significant change (*p* < 0.05) in Activities Balance Confidence, Reintegration of Normal Living Index, Prosthesis Evaluation Questionnaire scores, and 2-min walk test. By contrast, the activity-based parameters estimated from actimetry showed no statistically significant changes (*p* > 0.05). Associations between self-reported and functional outcomes and actimetry parameters were limited.

**Conclusions:**

Perceived and in-clinic outcome measures improved after short-term provision of an MPK for transfemoral amputees. However, PA did not change in this cohort of patients over the study period. More longitudinal studies are needed to characterize the impact of MPK provision on PA and societal participation.

## Introduction

Prolonged sedentary behavior is detrimental to physical and mental health and quality of life.^1^ Indeed, physical inactivity is responsible for one in 6 deaths in the United Kingdom and is estimated to cost the National Health Service (NHS) approximately £7.4 billion annually.^2,3^ Accordingly, the World Health Organization and governments have issued recommendations to reduce sedentary time.^4,5^ After lower limb amputation (LLA), many people become less active and lose independence.^6^ Therefore, there is an urgent need to promote physical activity (PA) among individuals with LLA and address societal barriers and facilitators to PA participation.

Individuals with LLA experience functional impairments, such as impaired balance and stability, and increased risk of falling.^7^ Intelligent prosthetic components such as microprocessor knees (MPKs) represent a technological advance in overcoming these challenges.^8,9^ Microprocessor knees are artificial knee joints that control both swing and stance phase of the user's gait. However, because of their high costs, MPKs may not be routinely available to individuals with LLA. Microprocessor knees were developed for individuals with a high functional mobility level, but in recent years, systematic reviews have identified their benefit for prosthesis users with lower mobility grades.^10^

Several studies have demonstrated the ability of MPKs to reduce the risk of falls and improve balance and safety. In addition, they have revealed increased mobility during overground walking, ambulation on uneven terrains, and other activities of daily living, when compared with non-MPK (nMPK) joints.^11,12^ These evaluations are mainly based on self-reported scales and questionnaires, for example, Activities Balance Confidence (ABC) scale, Reintegration of Normal Living Index (RNLI), and the Prosthesis Evaluation Questionnaire (PEQ), which assess a person's perceived ability to complete functional activities, ability to return to community participation, and satisfaction with their prosthesis, respectively. Many studies also use functional tests, for example, 2-min walk test (2MWT) and timed up and go (TUG), which assess basic function and mobility such as velocity, transfers, balance, and falls risk.^13^

However, a limited number of studies investigated improvements pre- and post-MPK provision using objective monitoring of daily activity derived from actimetry systems, which may provide further information from community-based PA parameters.^14-17^ In addition, previous studies have been limited in corroborating patient-reported measures with more objective assessments of function. A study from the authors^18^ investigated satisfaction, social engagement, and PA in a small cohort of lower limb prosthesis users in Cambodia and compared self-reported and objective measures. An association was observed between measured activity levels and socket satisfaction. Indeed, participants who were more active wore their prosthesis for longer and were more satisfied with socket fit. Another study of interest^14^ reported an improvement in function and mobility and a reduction in falls in individuals with low functional mobility after provision of MPK. In particular, a 20% increase in active time was documented with the actimetry system, which supported the improvement in the PEQ questionnaire and an increase in walking distance after the 2MWT. By contrast, in a similar cohort of people with transfemoral amputation, Theeven et al^15^ observed no significant improvements in mobility post-MPK provision.

Thus, with conflicting evidence, there is a need for further investigation. Therefore, this study aims to evaluate whether the provision of an MPK increases the level of AP in individuals with transfemoral amputation after a 4-week trial. Secondary analysis was conducted to investigate whether patient-reported outcomes and in-clinic functional activity test results correlated with objective PA parameters derived from actimetry systems.

## Methods

This study was a case-controlled pre- and post-MPK provision observational cohort study of people with transfemoral amputation presenting to 2 different prosthetic clinics in the South of England. Written informed consent was obtained from each patient before data collection, and ethics approval was granted by the Health Research Authority (IRAS number: 261545). During the study, self-reported outcomes and objective parameters were collected before and after the provision of an MPK. Patients were eligible for this study if they met the criteria for an NHS MPK trial.^19^ The eligibility criteria are set by NHS England and are detailed in Table 1. In addition, for uniformity, people with hip disarticulation were excluded. Furthermore, participants were excluded if they were involved in another trial or did not have sufficient English to give informed consent. The study designed involved a 4-week trial with the MPK device before follow-up assessment. If at the end of this period, the participant and the clinical team agreed, the MPK became the definitive prosthesis.

**Table 1.**
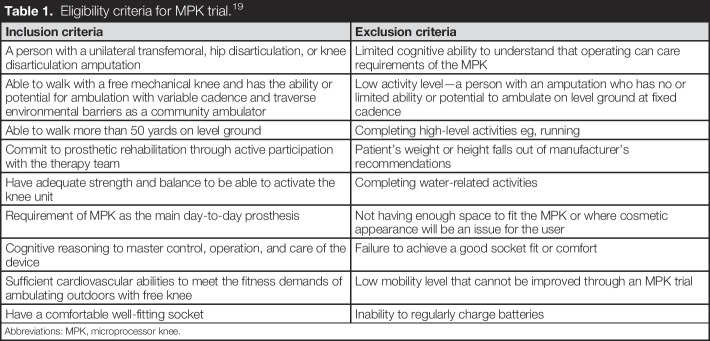
Eligibility criteria for MPK trial.^19^

Inclusion criteria	Exclusion criteria
A person with a unilateral transfemoral, hip disarticulation, or knee disarticulation amputation	Limited cognitive ability to understand that operating can care requirements of the MPK
Able to walk with a free mechanical knee and has the ability or potential for ambulation with variable cadence and traverse environmental barriers as a community ambulator	Low activity level—a person with an amputation who has no or limited ability or potential to ambulate on level ground at fixed cadence
Able to walk more than 50 yards on level ground	Completing high-level activities eg, running
Commit to prosthetic rehabilitation through active participation with the therapy team	Patient's weight or height falls out of manufacturer's recommendations
Have adequate strength and balance to be able to activate the knee unit	Completing water-related activities
Requirement of MPK as the main day-to-day prosthesis	Not having enough space to fit the MPK or where cosmetic appearance will be an issue for the user
Cognitive reasoning to master control, operation, and care of the device	Failure to achieve a good socket fit or comfort
Sufficient cardiovascular abilities to meet the fitness demands of ambulating outdoors with free knee	Low mobility level that cannot be improved through an MPK trial
Have a comfortable well-fitting socket	Inability to regularly charge batteries

Abbreviations: MPK, microprocessor knee.

### MPK trial protocol

Data collection included 3 separate prosthetic clinic visits which were part of usual care during an MPK trial. During the first visit, participants were asked to perform a 2MWT and TUG with their current prosthesis (nMPK). They were also asked to complete 3 self-reported questionnaires, namely, ABC scale, RNLI, and PEQ. On this occasion, an activity monitor (activPAL 4; PAL Technologies, Ltd, Glasgow, United Kingdom) was placed onto the central anterior aspect of their socket using an adhesive dressing and used to collect activity data for a period of 7 consecutive days. The activPAL accelerometers have been shown to have high reporting accuracy of step count and walking, sit-to-stand transitions, and sedentary time in community-dwelling older adults.^20,21^ It also has acceptable levels of accuracy for measuring walking time in individuals using a prosthesis.^22^

After 2–4 weeks, participants returned into clinic where they were fitted with an MPK and a prosthetic foot, according to the manufacturer's fitting guidelines. After a 4-week trial period recommended as part of NHS MPK policy provision,^19^ they returned into clinic to repeat the 2MWT and TUG test and complete the self-reported questionnaires. If the participants' trial period was successful according to the NHS MPK policy requirements,^19^ the participants were able to keep the MPK as their definitive prosthesis and the activity monitor was secured onto their socket to collect the activity parameters for 7 consecutive days. If the trial was unsuccessful and participants wished to return to the nMPK, their involvement in the study ceased.

### Data processing and analysis

The activity monitor sampled with a frequency of 20 Hz. Data were retrieved for analysis using the proprietary activPAL software (V7 2.32). Activity parameters included active time, for example, standing, walking, and cycling, and sedentary time, for example, sitting or lying, upright time, number of sit to stand events, and number of steps.

Statistical analysis was performed using IBM SPSS statistics V22 (IBM Corp, Armonk, NY). The PEQ subscales were combined to produce a total score. Data were examined for normality using Shapiro–Wilk tests. Parametric statistics (mean ± standard deviation (SD)) were found to be appropriate for analysis of ABC, RNLI, PEQ, 2MWT, and TUG, whose difference pre- and post-MPK provision was examined using a paired *t* test. Parametric statistics were also shown to be appropriate for actimetry data, with paired *t* tests used to compare between time points of evaluation. Pearson correlation was used to assess the relationship between subjective and objective parameters. For all outcomes, the statistical significance level was set at the 5% level (*p* ≤ 0.05).

## Results

### Patients

In total, 29 participants were recruited over an 18-month period, including 23 males and 5 females aged between 26 and 87 years (mean = 60 years). However, follow-up visits were not carried out for 11 patients, because of access restrictions created by the COVID-19 pandemic. Therefore, these participants were removed from the cohort, leaving 14 participants (12 males and 2 females). They had a mean age of 57 years (range 41–78 years), with the number of years since amputation ranging between 1 and 44 (mean = 22), and an average number of comorbidities of 2 (range 0–6). Main reasons for amputation were trauma, infection, tumor, and peripheral vascular disease. Nine were provided with a C-leg MPK, which was the most provided. Table 2 summarizes the demographic data of the patients included in the analysis. Table 2 shows a positive percentage difference to indicate an improvement. It is worth noting that the magnitude of changes in both self-reported and functional outcomes was varied across the cohort.

**Table 2.**
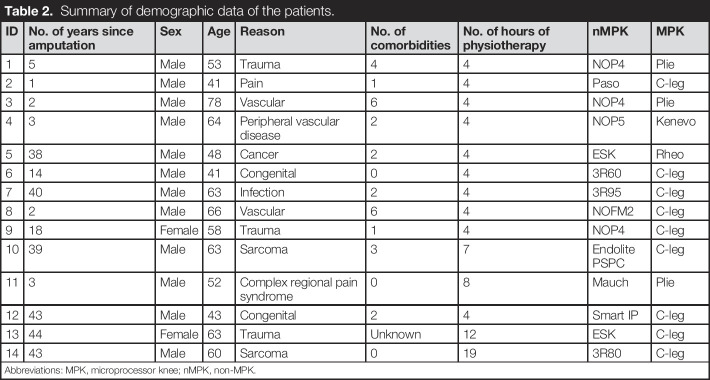
Summary of demographic data of the patients.

ID	No. of years since amputation	Sex	Age	Reason	No. of comorbidities	No. of hours of physiotherapy	nMPK	MPK
1	5	Male	53	Trauma	4	4	NOP4	Plie
2	1	Male	41	Pain	1	4	Paso	C-leg
3	2	Male	78	Vascular	6	4	NOP4	Plie
4	3	Male	64	Peripheral vascular disease	2	4	NOP5	Kenevo
5	38	Male	48	Cancer	2	4	ESK	Rheo
6	14	Male	41	Congenital	0	4	3R60	C-leg
7	40	Male	63	Infection	2	4	3R95	C-leg
8	2	Male	66	Vascular	6	4	NOFM2	C-leg
9	18	Female	58	Trauma	1	4	NOP4	C-leg
10	39	Male	63	Sarcoma	3	7	Endolite PSPC	C-leg
11	3	Male	52	Complex regional pain syndrome	0	8	Mauch	Plie
12	43	Male	43	Congenital	2	4	Smart IP	C-leg
13	44	Female	63	Trauma	Unknown	12	ESK	C-leg
14	43	Male	60	Sarcoma	0	19	3R80	C-leg

Abbreviations: MPK, microprocessor knee; nMPK, non-MPK.

### Patient-reported and functional outcomes

**Figure 1.**
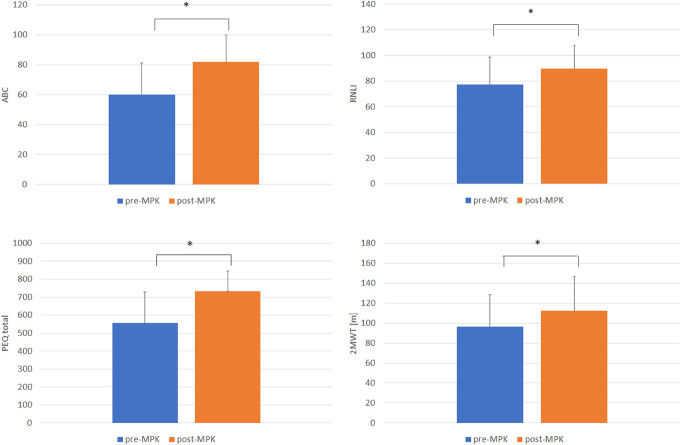
Mean values of (top left) ABC, (top right) RNLI, (bottom left) PEQ, and (bottom right) 2MWT, collected with the nMPK (blue) and after the provision of the MPK (orange). Error bars indicate the standard deviation, and * indicates a statistically significant difference (*p* < 0.05). 2MWT, 2-min walk test; ABC, Activities Balance Confidence; PEQ, Prosthesis Evaluation Questionnaire; MPK, microprocessor knee; nMPK, non-MPK; RNLI, Reintegration of Normal Living Index.

Self-reported scores, 2MWT, and TUG outcomes showed a general improvement in most of the patients after the provision of the MPK (Table 3). A statistically significant increase (*p* < 0.05) was observed in all the self-reported scores and 2MWT after the provision of the MPK (Figure 1). By contrast, TUG showed similar values (mean values, pre-MPK = 29.8 (±11.1); post-MPK = 28.9 (±15.9); *p* > 0.05). On closer inspection, 10 of 14 patients reported a balance confidence score of at least 20% higher when using the MPK, with the majority also showing an improvement greater than 10% in the RNLI index. However, it is worth noting that patient 14 reported scores of 9.5 and 9.8, when using the nMPK and MPK, respectively, showing a poor reintegration within the normal activities of daily living. Results from the 2MWT showed that 86% of the patients were able to cover a longer distance on their MPK (mean = 112.3 ± 34.4 m) than the nMPK (mean = 96.6 ± 32.1 m). Timed up and go showed lower values with the MPK assessment, denoting a reduction in the time to perform the time up and go compared with nMPK.

**Table 3.**
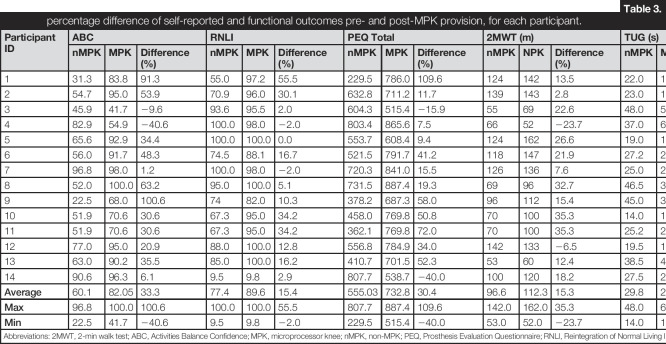
Absolute values and percentage difference of self-reported and functional outcomes pre- and post-MPK provision, for each participant.

Participant ID	ABC			RNLI			PEQ Total			2MWT (m)			TUG (s)		
	nMPK	MPK	Difference (%)	nMPK	MPK	Difference (%)	nMPK	MPK	Difference (%)	nMPK	NPK	Difference (%)	nMPK	MPK	Difference (%)
1	31.3	83.8	91.3	55.0	97.2	55.5	229.5	786.0	109.6	124	142	13.5	22.0	18.0	20.0
2	54.7	95.0	53.9	70.9	96.0	30.1	632.8	711.2	11.7	139	143	2.8	23.0	15.0	42.1
3	45.9	41.7	−9.6	93.6	95.5	2.0	604.3	515.4	−15.9	55	69	22.6	48.0	50.0	−4.1
4	82.9	54.9	−40.6	100.0	98.0	−2.0	803.4	865.6	7.5	66	52	−23.7	37.0	68.0	−59.0
5	65.6	92.9	34.4	100.0	100.0	0.0	553.7	608.4	9.4	124	162	26.6	19.0	16.0	17.1
6	56.0	91.7	48.3	74.5	88.1	16.7	521.5	791.7	41.2	118	147	21.9	27.2	24.2	11.7
7	96.8	98.0	1.2	100.0	98.0	−2.0	720.3	841.0	15.5	126	136	7.6	25.0	25.0	0.0
8	52.0	100.0	63.2	95.0	100.0	5.1	731.5	887.4	19.3	69	96	32.7	46.5	35.0	28.2
9	22.5	68.0	100.6	74	82.0	10.3	378.2	687.3	58.0	96	112	15.4	45.0	32.0	33.8
10	51.9	70.6	30.6	67.3	95.0	34.2	458.0	769.8	50.8	70	100	35.3	14.0	12.8	8.3
11	51.9	70.6	30.6	67.3	95.0	34.2	362.1	769.8	72.0	70	100	35.3	25.2	21.2	17.0
12	77.0	95.0	20.9	88.0	100.0	12.8	556.8	784.9	34.0	142	133	−6.5	19.5	18.5	5.0
13	63.0	90.2	35.5	85.0	100.0	16.2	410.7	701.5	52.3	53	60	12.4	38.5	45.6	−17.0
14	90.6	96.3	6.1	9.5	9.8	2.9	807.7	538.7	−40.0	100	120	18.2	27.5	22.8	18.7
**Average**	60.1	82.05	33.3	77.4	89.6	15.4	555.03	732.8	30.4	96.6	112.3	15.3	29.8	28.9	8.7
**Max**	96.8	100.0	100.6	100.0	100.0	55.5	807.7	887.4	109.6	142.0	162.0	35.3	48.0	68.0	42.1
**Min**	22.5	41.7	−40.6	9.5	9.8	−2.0	229.5	515.4	−40.0	53.0	52.0	−23.7	14.0	12.8	−59.0

Abbreviations: 2MWT, 2-min walk test; ABC, Activities Balance Confidence; MPK, microprocessor knee; nMPK, non-MPK; PEQ, Prosthesis Evaluation Questionnaire; RNLI, Reintegration of Normal Living Index; TUG, timed up and go.

### Objective parameters from actimetry

**Figure 2.**
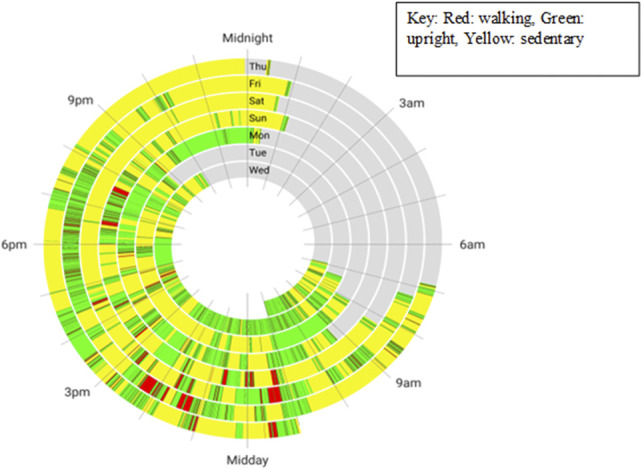
Pin wheel view of data estimated from the actimetry for P#1.

A typical activity monitoring period for one patient is represented in Figure 2. The pin wheel depicts the activities that are monitored daily, over the 1-week study period.

Table 4 summarizes the absolute values and the percentage difference of the parameters estimated from the actimetry between pre- and post-MPK assessment time points. The number of steps decreased in 10 participants when using the MPK, 7 of whom reported a decrement ≥10%. The same participants showed a decrease in their overall active time (−5% to −40%).

**Table 4.**
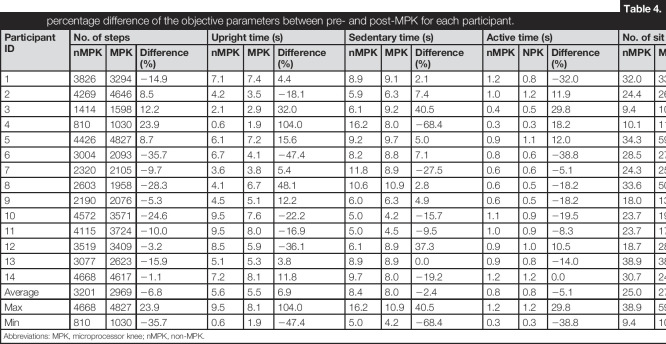
Absolute values and percentage difference of the objective parameters between pre- and post-MPK for each participant.

Participant ID	No. of steps			Upright time (s)			Sedentary time (s)			Active time (s)			No. of sit to stand events		
	nMPK	MPK	Difference (%)	nMPK	MPK	Difference (%)	nMPK	MPK	Difference (%)	nMPK	NPK	Difference (%)	nMPK	MPK	Difference (%)
1	3826	3294	−14.9	7.1	7.4	4.4	8.9	9.1	2.1	1.2	0.8	−32.0	32.0	33.7	5.2
2	4269	4646	8.5	4.2	3.5	−18.1	5.9	6.3	7.4	1.0	1.2	11.9	24.4	26.0	6.4
3	1414	1598	12.2	2.1	2.9	32.0	6.1	9.2	40.5	0.4	0.5	29.8	9.4	10.0	6.0
4	810	1030	23.9	0.6	1.9	104.0	16.2	8.0	−68.4	0.3	0.3	18.2	10.1	11.0	8.1
5	4426	4827	8.7	6.1	7.2	15.6	9.2	9.7	5.0	0.9	1.1	12.0	34.3	59.6	53.9
6	3004	2093	−35.7	6.7	4.1	−47.4	8.2	8.8	7.1	0.8	0.6	−38.8	28.5	27.5	−3.6
7	2320	2105	−9.7	3.6	3.8	5.4	11.8	8.9	−27.5	0.6	0.6	−5.1	24.3	25.3	4.0
8	2603	1958	−28.3	4.1	6.7	48.1	10.6	10.9	2.8	0.6	0.5	−18.2	33.6	50.2	39.6
9	2190	2076	−5.3	4.5	5.1	12.2	6.0	6.3	4.9	0.6	0.5	−18.2	18.0	13.0	−32.3
10	4572	3571	−24.6	9.5	7.6	−22.2	5.0	4.2	−15.7	1.1	0.9	−19.5	23.7	19.0	−21.9
11	4115	3724	−10.0	9.5	8.0	−16.9	5.0	4.5	−9.5	1.0	0.9	−8.3	23.7	17.8	−28.4
12	3519	3409	−3.2	8.5	5.9	−36.1	6.1	8.9	37.3	0.9	1.0	10.5	18.7	28.5	41.5
13	3077	2623	−15.9	5.1	5.3	3.8	8.9	8.9	0.0	0.9	0.8	−14.0	38.9	38.5	−1.0
14	4668	4617	−1.1	7.2	8.1	11.8	9.7	8.0	−19.2	1.2	1.2	0.0	30.7	24.6	−22.1
Average	3201	2969	−6.8	5.6	5.5	6.9	8.4	8.0	−2.4	0.8	0.8	−5.1	25.0	27.5	4.0
Max	4668	4827	23.9	9.5	8.1	104.0	16.2	10.9	40.5	1.2	1.2	29.8	38.9	59.6	53.9
Min	810	1030	−35.7	0.6	1.9	−47.4	5.0	4.2	−68.4	0.3	0.3	−38.8	9.4	10.0	−32.3

Abbreviations: MPK, microprocessor knee; nMPK, non-MPK.

**Figure 3.**
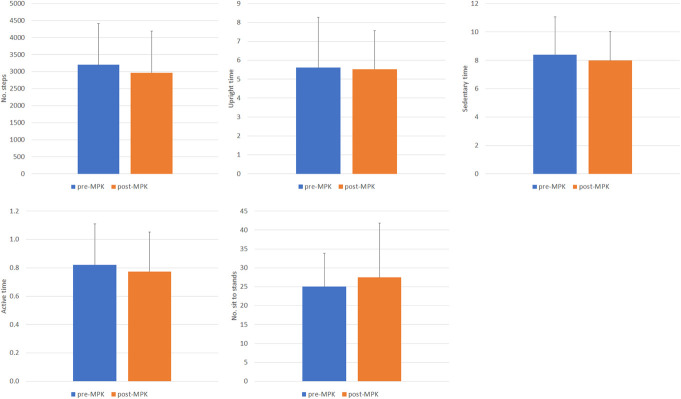
Mean values of number of steps (top left), upright time (top middle), sedentary time (top right), active time (bottom left), and number of sit to stand events (bottom middle), collected with the nMPK (blue) and after the provision of the MPK (orange). Error bars indicate the standard deviation. MPK, microprocessor knee; nMPK, non-MPK.

Figure 3 reveals the participants' mean values across the objective parameters, collected when using the nMPK and after the provision of the MPK. There were a high degree of intersubject variability and limited mean difference between time points, which resulted in no statistically significant (*p* > 0.05) changes in any of the activity-based parameters.

### Association between self-reported and functional outcomes with objective parameters

When self-reported and functional outcomes were compared with the parameters estimated from the actimetry sensor, the resulting associations yielded a few statistically significant correlations (Table 5). No specific trends were observed when nMPK and MPK correlations were compared.

**Table 5.**
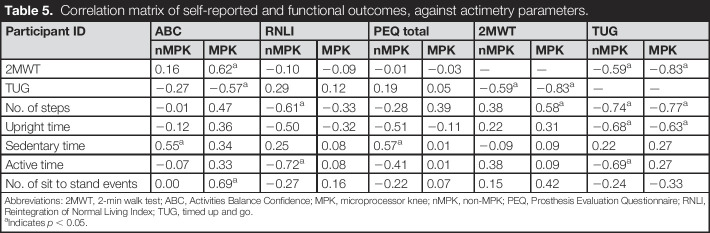
Correlation matrix of self-reported and functional outcomes, against actimetry parameters.

Participant ID	ABC		RNLI		PEQ total		2MWT		TUG	
	nMPK	MPK	nMPK	MPK	nMPK	MPK	nMPK	MPK	nMPK	MPK
2MWT	0.16	0.62^a^	−0.10	−0.09	−0.01	−0.03	—	—	−0.59^a^	−0.83^a^
TUG	−0.27	−0.57^a^	0.29	0.12	0.19	0.05	−0.59^a^	−0.83^a^	—	—
No. of steps	−0.01	0.47	−0.61^a^	−0.33	−0.28	0.39	0.38	0.58^a^	−0.74^a^	−0.77^a^
Upright time	−0.12	0.36	−0.50	−0.32	−0.51	−0.11	0.22	0.31	−0.68^a^	−0.63^a^
Sedentary time	0.55^a^	0.34	0.25	0.08	0.57^a^	0.01	−0.09	0.09	0.22	0.27
Active time	−0.07	0.33	−0.72^a^	0.08	−0.41	0.01	0.38	0.09	−0.69^a^	0.27
No. of sit to stand events	0.00	0.69^a^	−0.27	0.16	−0.22	0.07	0.15	0.42	−0.24	−0.33

Abbreviations: 2MWT, 2-min walk test; ABC, Activities Balance Confidence; MPK, microprocessor knee; nMPK, non-MPK; PEQ, Prosthesis Evaluation Questionnaire; RNLI, Reintegration of Normal Living Index; TUG, timed up and go.

a Indicates *p* < 0.05.

Participants with higher individual confidence during ambulatory activities with the MPK (ABC score) covered a higher distance in the 2MWT (*r* = 0.62, *p* < 0.05) and achieved a lower time in the TUG (*p* = −0.57, *p* < 0.05). In addition, there was a significant positive correlation between the ABC score and the number of sit to stand events (*r* = 0.69, *p* < 0.05). By contrast, no statistically significant associations were found in their nMPK counterparts. The number of steps statistically significantly correlated with both 2MWT and TUG (*p* < 0.05), revealing the higher the number of steps performed, the higher the distance travelled in the 2MWT. Similarly, the higher the number of steps, upright time, and active time, the lower the time to complete the TUG test, with both nMPK and MPK.

## Discussion

Our study evaluated the short-term changes in perceived and observed function after the provision of MPK components in people with transfemoral amputation from 2 different prosthetic centers. Our results indicate that the provision of an MPK improved balance confidence, patient satisfaction, mobility, and function, reflected by statistically significant improvements in ABC, RNLI, and PEQ scores. In addition, in-clinic functional tests also improved, for example, 2MWT (Figure 1). These findings are comparable with previous studies assessing changes in function, satisfaction, balance confidence, and mobility in individuals with transfemoral amputation who are provided an MPK.^14,16,23,24^ However, MPK acclimatization period and time post-MPK prescription differed between studies.^14^ There were no statistically significant changes observed in PA-based parameters estimated from actimetry. This demonstrates some differences between patient-reported outcomes and PA parameters such as step count and could be indicative that these monitoring parameters may not be sensitive to detect functional changes within the amputee cohort. Further longitudinal studies are required to better assess the role of activity monitoring in prosthetic outcomes.

Literature reveals several studies^25-27^ describing a strong association between in-clinic physical performance measures, for example, 2MWT, and level of activity. Our results only partially support this finding for individuals with LLA. Indeed, the association between 2MWT and activity-based parameters (Table 5) showed only a positive correlation with the number of steps when using the MPK, despite this measure being lower when compared with pre-MPK. Our findings showed no specific trends between nMPK and MPK when the self-reported and functional outcomes were compared against the parameters estimated from the actimetry sensor. It is worth noting that the TUG had several significant correlations with the actimetry data, including steps, upright time, and active time (Table 5). This has also been demonstrated in other studies, where TUG was associated with step count.^28^

The psychometric properties of actimetry data, such as step count, should be considered. The reliability and validity of the activPAL3 for measuring physical behaviors in older adults and adults with unilateral limb loss have been reported.^29-31^ While there is consensus that the activPAL is a reliable measurement tool in adults with limb loss in a laboratory setting, further evaluation is needed to assess whether similar evidence is found in free-living activity and sedentary contexts. Our data revealed that the number of daily steps of our cohort ranged between 1030 and 4827, which was observed to be wider than that in previous studies. For example, Pepin et al^32^ reported approximately 3000–4000 steps/day. In this study, 4/14 patients showed steps between 3000 and 4000 and only 3/14 showed steps greater than 4000. The patients recruited to this study revealed an increased active time with the MPK use, but they also showed an increased sedentary time. This may indicate that participants worn their MPK longer, and this could be reflected in higher activity-related confidence community participation and prosthetic satisfaction. The interpretation of meaningful number or change in step count in individuals with limb loss is yet to be defined, limiting the understanding of how step count may inform clinical decision making.

## Limitations

This study includes some limitations. In particular, the short-term nature of the 4-week trial period during which the patients were in the process of learning how to use the MPK will have limited potential for significant change in activity and participation. Indeed, this period might be relatively short for some individuals to fully learn and trust the device and therefore increase their level of activity. Longer follow-up would have been ideal to assess the long-term effect of the MPK. Not all patients used the same MPK, prosthetic feet, or suspension method, and therefore, it is not known whether these factors influence their function and mobility. Patient-reported measures were also used as part of routine care. Therefore, participants might have been biased toward the MPK to secure its use. In addition, the criterion validity of several accelerometer systems has been defined in step counting and distance measurement in adult populations.^33^ However, it is noted that there may be differences in the absolute values between devices.^34^ This is mitigated in our study using the pre- and postdesign with the same actimetry system. However, it might limit the generalizability of our findings to other commercial activity monitors.

Further research is needed to complete a larger prospective study, objectively measuring a range of patient-reported outcomes^35^ and activity to assess whether the provision of the MPK increases function and quality of life in individuals with limb loss. This would include a longer acclimatization period to allow full adaptation to MPK and habit change and a long-term follow-up to assess if any change is sustained over time. This could provide more robust data, and in-depth assessments could be made regarding other factors including the influence of the type of MPK used.

## Clinical Implications

The patients' self-reported improvements and significant changes in clinical assessments of physical function suggest that the MPK provided improved perceived and in-clinic outcomes in the short term. This cannot be generalized with the study population included in this evaluation, and further longitudinal cohort studies are needed to evaluate the benefit of MPK provision. The daily step outcomes assessed using actimetry showed no change pre- and post-MPK. However, comorbidities may be associated with daily step counts after amputation and should be included as a confounder in any future studies.^36^ Further exploration of PA monitoring as an outcome from successful rehabilitation should be explored, where more nuanced assessment of different categories of activity and movement quality maybe required to explore the full benefits of different prosthetic components.^17^

## Conclusion

Provision of an MPK improved self-reported mobility, function, and prosthetic satisfaction. This highlights an improved outcome after MPK provision within a relatively short period of follow-up observed in this study. There was a large degree of intra- and intersubject variability in the activity-based parameters monitored over a 7-day period, pre- and post-MPK provision. Future studies are needed to explore the role of activity monitoring and step count in assessing outcomes in individuals with lower limb loss. This should include patient-perceived benefit and an observed change in function.

## Funding

The author(s) disclosed receipt of the following financial support for the research, authorship, and/or publication of this article: This work was supported by the Engineering and Physical Sciences Research Council (EPSRC) (Ref EP/R014213/1).

## Declaration of conflicting interest

The author(s) disclosed no potential conflicts of interest with respect to the research, authorship, and/or publication of this article.

## Supplemental material

No supplemental digital content is available in this article.
